# Comparative metabolomics in primates reveals the effects of diet and gene regulatory variation on metabolic divergence

**DOI:** 10.1038/srep05809

**Published:** 2014-07-28

**Authors:** Ran Blekhman, George H. Perry, Sevini Shahbaz, Oliver Fiehn, Andrew G. Clark, Yoav Gilad

**Affiliations:** 1 Department of Genetics, Cell Biology, and Development, University of Minnesota, Minneapolis, MN, 55455; 2 Department of Ecology, Evolution, and Behavior, University of Minnesota, St. Paul, MN, 55108; 3 Department of Biology, Penn State University, University Park, PA, 16802; 4 UC Davis Genome Center, University of California, Davis, CA, 95616; 5 Department of Molecular Biology and Genetics, Cornell University, Ithaca, NY, 14853; 6 Department of Human Genetics, University of Chicago, Chicago, IL, 61637

## Abstract

Human diets differ from those of non-human primates. Among few obvious differences, humans consume more meat than most non-human primates and regularly cook their food. It is hypothesized that a dietary shift during human evolution has been accompanied by molecular adaptations in metabolic pathways. Consistent with this notion, comparative studies of gene expression levels in primates have found that the regulation of genes with metabolic functions tend to evolve rapidly in the human lineage. The metabolic consequences of these regulatory differences, however, remained unknown. To address this gap, we performed a comparative study using a combination of gene expression and metabolomic profiling in livers from humans, chimpanzees, and rhesus macaques. We show that dietary differences between species have a strong effect on metabolic concentrations. In addition, we found that differences in metabolic concentration across species are correlated with inter-species differences in the expression of the corresponding enzymes, which control the same metabolic reaction. We identified a number of metabolic compounds with lineage-specific profiles, including examples of human-species metabolic differences that may be directly related to dietary differences.

Human diets, whether those of traditional hunter-gatherer societies, subsistence agriculturalists, or modern industrialized societies, are different from the diets of all non-human primates. This difference can be understood as a consequence of a series of major dietary shifts in the evolutionary history of the human lineage, including meat scavenging and hunting behavior, the controlled use of fire and cooking, plant and animal domestication, and most recently, the widespread use of chemical additives^1^. In contrast to the diets of most modern human populations, which incorporate many foods including meat but are often starch-based (in agricultural subsistence societies: 50–70% of total calories from starch^2^), chimpanzees are predominantly frugivorous and ingest relatively little starch and meat compared to humans^3^. This extreme dietary shift in recent human history has been proposed as a major driving force of human evolution. For example, the advent of cooking, which has led to consumption of a higher quality diet, is suggested to underlie a concurrent decrease in gut size and increase in brain size, and perhaps is also a driver of certain shifts in human social behavior^4^,^5^.

Some of the dietary changes during human evolution were likely accompanied by corresponding molecular adaptations. In particular, one might expect human-specific adaptations in metabolic pathways. One remarkable example of dietary adaptation accompanied by positive selection on a metabolic pathway is lactase persistence, the ability of humans to digest lactose as adults^6^,^7^. This trait, which is thought to be unique to humans among mammals, is facilitated by substitutions in regulatory regions near the *LCT* gene, which encodes the lactase enzyme that metabolizes lactose^6^,^8^,^9^. Adaptive changes in gene regulation, such as the ones described for *LCT*, have long been thought to play a major role in human evolution.

Recent comparative studies have found large numbers of differences in gene regulation between primate species in all tissues examined to date^10^,^11^,^12^,^13^,^14^. Interestingly, genes involved in metabolism were found to be enriched among genes whose regulation may have evolved under positive selection in the human liver (using as background for the enrichment analysis the particular set of genes expressed in the liver in the first place^13^,^14^). Moreover, genes involved in metabolism are also enriched among genes whose promoters show evidence for positive selection in humans^15^. These results suggest an important role for gene regulatory evolution in dietary and metabolic adaptation, and prompt a further investigation of inter-primate gene expression differences in the context of metabolic differences between species.

Here, we employed gas chromatography/time-of-flight mass spectrometry (GC-TOF MS) to quantify the levels of hundreds of nutrients and metabolic compounds in livers from humans, chimpanzees (*Pan troglodytes*), and rhesus macaques (*Macaca mulatta*), using samples from six individuals from each species, from which we previously collected gene expression data (from the same individuals)^14^. We compared the liver metabolic profiles of the three species and obtained a partial view of the effects of inter-species dietary differences on liver metabolism. Moreover, by considering the metabolic profiles along with gene expression measurements from the same samples, we gained insight into the interplay between the regulation of enzymes and between-species metabolic and dietary differences.

## Results

We used GC-TOF MS to measure metabolite concentrations in liver samples from six human, six chimpanzee, and six rhesus macaque individuals. We performed three technical replicates for each sample, for a total of 54 measurements. The identities of all metabolites were validated through a multi-tiered matching algorithm in our in-house BinBase database system^16^ using retention index information in addition to mass spectral matching, unique ion characteristics, peak purity, and signal/noise metadata for final metabolite reporting (Supplementary Methods).

Overall we measured the concentration of 399 metabolites in all samples, of which 177 were known and had an associated name. This number of measured metabolites is roughly 20% greater than usually found in human body fluids^17^, reflecting the higher metabolic activity and complexity in liver tissues. After normalization, we performed quality control by using a principal component analysis and inter-correlation analysis of the data, and excluded three outlier replicates. In addition, we regressed out two principal components that represented non-biological artifacts, such as an apparent batch effect (see Supplementary Methods). The complete dataset used in all following analyses is available in Supplementary Table S1.

### Differences in metabolite levels between species

Our main goal was to identify differences in metabolite concentrations among species. To do so, we used a linear mixed-effects model, with a fixed effect for species, to analyze the normalized comparative metabolomics data (see Methods). Using likelihood-ratio tests within the framework of the linear model, we identified metabolites with significant differences in concentration between each pair of species (FDR < 0.05). We thus classified the concentration of 122, 96, and 29 metabolites as significantly different between human-chimpanzee, human-rhesus macaque, and chimpanzee-rhesus macaque, respectively (see Supplementary Fig. S3). Overall, the concentrations of 129 metabolites differ between human and either of the other two species, yet show no evidence for differences between chimpanzee and rhesus macaque. The number of species-specific changes in metabolite concentration (Supplementary Fig. S2) is much smaller for chimpanzee (62) and rhesus macaque (36).

**Figure 1:**
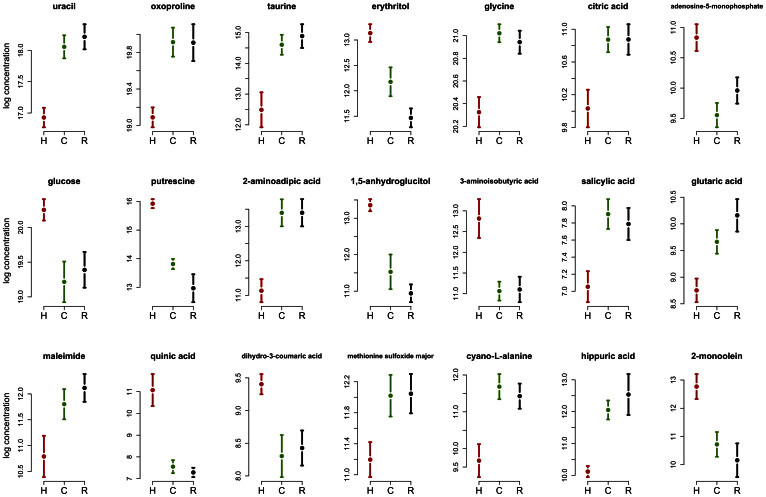
Known metabolites with human-specific levels. Metabolite levels (*y*-axis, log-scale) in human (red), chimpanzee (green), and rhesus macaque (black). Error bars represent the ±SE within each species.

In order to characterize further the metabolites that show human-specific changes in concentration levels, we first focused on metabolites for which names have been identified in the KEGG database (http://www.kegg.jp18). We defined a human-specific metabolic pattern as a significant difference in concentration level between human and both chimpanzee and rhesus macaque (FDR < 0.05), while showing no evidence for difference between the two non-human primates (FDR > 0.1). We found 21 metabolites with concentration levels consistent with this inter-species pattern, which also have a known name and record in KEGG (shown in Fig. 1).

We expect that a number of the human-specific metabolite concentrations are likely the result of modern human-specific diet (namely, these may reflect environmental effects on metabolic profiles). For example, erythritol, which has a significantly higher concentration in humans compared to the non-human primates, is sugar alcohol that is often used as a food sweetener. In addition, glucose, mono olein (palm oil), and quinic acid (a metabolite obtained from coffee beans) all have higher concentration in humans, consistent with their higher abundance in a industrialized agricultural society human diet.

Other examples may reflect more ancient shifts in diet during human evolution. For example, salicylic acid, an important plant hormone^19^, is found at significantly lower concentration in the human liver compared with the livers of the non-human primates. Major dietary sources for salicylic acid include plants and fruits, and it is especially high in berries, as well as in some herbs and spices. On the other hand, cereals, meat, fish, poultry, eggs, and dairy products contain little to no salicylic acid^20^. This result is therefore consistent with differences in diet among the three primate species, as well as with previous observations that serum salicylic acid level is higher in vegetarians compared to non-vegetarians^21^.

### Inter-species differences in metabolic pathways

Since it is difficult to make sense of inter-species differences in individual metabolites, we aggregated the evidence that support human-specific concentrations of compounds in metabolic pathways. To do so, we extracted pathway information from KEGG and examined the combined evidence for species-specific changes in the metabolic pathway by considering the P-values associated with tests for inter-species differences in individual metabolite concentration (Supplementary Methods). The pathways showing the strongest evidence for human-specific metabolic patterns (Supplementary Table S3) include lysine degradation, arginine and proline metabolism, phenylalanine metabolism, phenylalanine, tyrosine and tryptophan biosynthesis, taurine and hypotaurine metabolism, cyanoamino acid metabolism, glutathione metabolism, and primary bile acid biosynthesis. It is not always clear how these pathways may be related to human-specific diets, but primary bile acid biosynthesis is a metabolic pathway whose primary function is to facilitate processing of dietary fat. This result is consistent with a human diet that is richer in meat and fat compared to the diet of the two non-human primates^3^.

**Figure 2:**
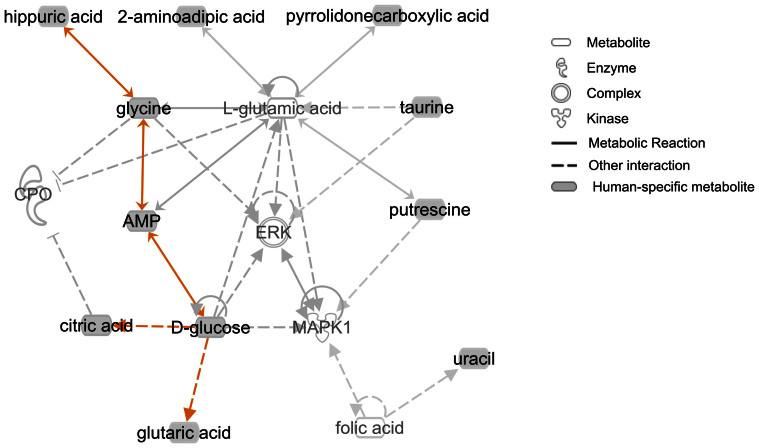
Interaction network enriched with human-specific metabolites. Shaded nodes indicate metabolites showing a human-specific concentration, with undashed lines connecting compounds involved in the same metabolic reaction. Orange lines are connecting human-specific metabolites. The interaction network was generated using the Ingenuity Pathway Analysis (IPA) tool.

In order to gain further insight on metabolic interactions among compounds that show human-specific levels, we used the Ingenuity pathway analysis tool (http://www.ingenuity.com/) to explore known interaction networks (Fig. 2). We found an interaction network that involves eleven of the 21 human-specific metabolites, with four of them (hippuric acid, glycine, AMP, and D-glucose) participating in the same metabolic reactions. The two most significantly enriched molecular functions among compounds in this network include Cell-To-Cell Signaling and Interaction, and Energy Production (Supplementary Table S4; P < 0.05). The two top canonical pathways enriched among metabolites in the network are Lysine Degradation and Insulin Receptor Signaling (Supplementary Table S5; P < 0.01). That some of the metabolites showing human-specific patterns are involved in the same reactions indicates that the inter-species pattern we observe may reflect shifts in particular metabolic phenotypes.

### Network-level view of differential concentration in metabolic reactions

Using metabolic pathway data from KEGG also allows us to examine changes in metabolite concentration between species in the context of the large-scale metabolic network. To do so, we examined the correlation between the number of reactions each metabolite is involved in, and the extent to which the concentration of the metabolite differs between species. As a first step, we summarized the number of reactions each metabolite is involved in and plotted the distribution of this quantity across metabolites (Supplementary Fig. S4). We found that although most metabolites are involved in fewer (<10) reactions, the distribution has a long tail as few metabolites are involved in many reactions. We therefore divided the metabolites into two groups, termed low- and high-connectivity metabolites, using 20 reactions as the cutoff (this is an arbitrary cutoff, but using a range of alternative cutoffs did not qualitatively change the subsequent result; see Supplementary Methods).

We then compared the distribution of likelihood ratio statistics for tests of inter-species differences in concentration between the high- and low-connectivity metabolite groups. We found that metabolites involved in many reactions are less likely to differ between human and either chimpanzee or rhesus macaque (Supplementary Fig. S5). This result has an intuitive explanation, as metabolites that are involved in more reactions may be less likely to be affected by perturbations in other parts of the network. In principle, this is similar to the observation that proteins that are involved in many cellular interactions evolve slower than proteins involved in only few interactions^22^. Our observations suggest an equivalent hypothesis may be true considering metabolic compounds; namely, that the concentration of metabolites is less likely to be different among species when they are involved in multiple reactions.

**Figure 3:**
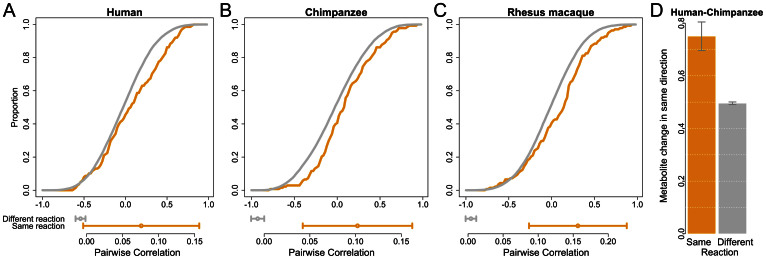
Metabolites involved in the same reactions change their levels in concert within and between species. Cumulative distribution (*y*-axis) of correlation coefficients across individuals (*x*-axis) for metabolites involved in the same reaction (orange) and different reactions (grey), within humans (A), chimpanzees (B), and rhesus macaques (C). Below each distribution we show the median pairwise correlation with error bars representing a 95% confidence interval calculated using bootstrapping (1000 resamplings); the difference in medians is significant in all species (human P = 6 × 10^−3^, chimpanzee P = 2 × 10^−3^, and rhesus macaque P < 10^−3^, using a permutation test; see SI Materials and Methods). (D) Proportion of metabolite pairs that change their levels in the same direction in human and chimpanzee, considering pairs where both metabolites are differentially concentrated between the species. The proportion is significantly different between metabolite pairs involved in the same reaction (orange) and different reactions (grey, P = 0.019 using a one-sided Fisher's exact test). See Supplementary Fig. S8 for a similar plot considering pairs where at least one of the metabolites is differentially concentrated between the species. Error bars represent 95% confidence intervals calculated using bootstrap resampling.

Next, we tested whether metabolites that are involved in the same reactions tend to have similar patterns across species. To examine this, we first calculated the pairwise correlation of metabolite concentrations across individuals within species. We then incorporated information from KEGG on metabolites that are involved in the same reaction, and examined the distribution of pairwise correlation values. We found that, in all three species, there is a significantly higher correlation between the concentrations of pairs of metabolites involved in the same reaction compared to metabolites involved in different reactions (see Fig. 3a–c, Supplementary Methods, Supplementary Fig. S6; permutation *P* < 6 × 10^−3^). This observation suggests that the rate of many metabolic reactions is tightly regulated across individuals.

To assess whether there is a similar correlated change in metabolite concentration across species, we focused on metabolites whose concentration significantly (FDR < 0.05) differs between human and chimpanzee, and examined the direction of change in concentration (i.e., which species has a higher level). We found that the concentrations of ~75% of metabolite pairs involved in the same reaction change in the same direction across species, compared to ~50% of pairs of metabolite not involved in the same reaction (Fig. 3d; *P* < 0.02 by Fisher's exact test). We observed the same pattern when we considered metabolite pairs where at least one metabolite shows significant difference in concentration between species (Supplementary Fig. S7, *P* = 0.015). These results suggest a coordinated inter-species change in the concentration levels of metabolites when they are involved in the same reaction.

A similar observation has been made considering gene expression and sequence divergence levels in yeast^23^. A possible explanation is that this coordinated shift could be a result of a change in a regulator; for example, an enzyme that changed its expression between species could drive a correlated change in concentration levels of the metabolites it controls. In the next section we test this hypothesis using a combination of metabolomics and gene expression data^14^.

### Enzyme expression and metabolite concentration differences between species

We combined liver gene expression data from the same eighteen samples^14^ with the metabolomics data and the reaction information from KEGG. We identified 1043 enzymes and 1629 enzyme-metabolite associations for which we have the corresponding gene expression and metabolomics data. To examine the correlation between regulatory and metabolomic changes we classified metabolites into two groups (either similar or different across species), based on the evidence that their concentration differs between human and chimpanzee. We then identified the enzymes that control reactions involving the metabolites, excluding enzymes that control reactions in both groups. We then considered the likelihood ratio tests for differences in gene expression levels between human and chimpanzee (as defined in Blekhman et al.^14^) for the transcripts that encode for the enzymes.

**Figure 4:**
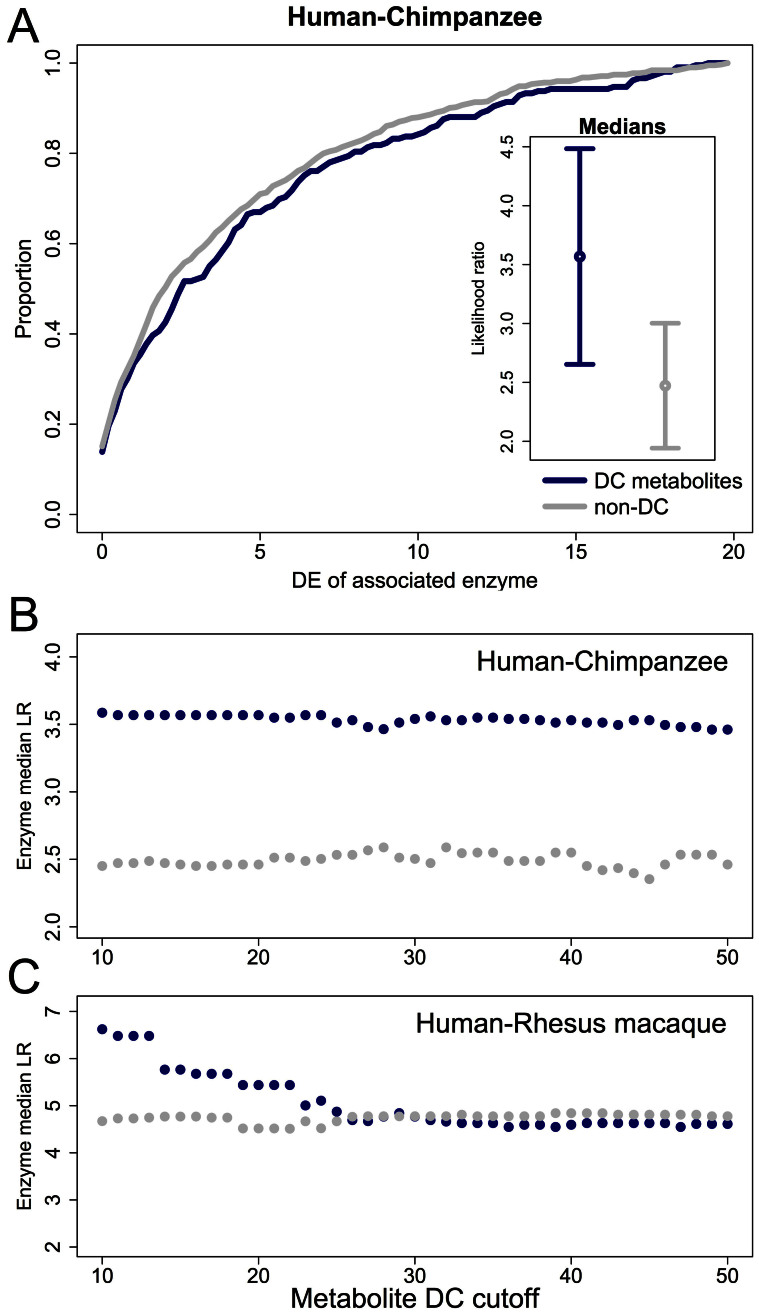
Metabolites that are differentially concentrated between species are more likely to be involved in reactions catalyzed by enzymes that are differentially expressed between species. (A) Cumulative distribution of likelihood ratios (LR) for differential expression (DE, *x*-axis) for enzymes associated with differentially concentrated (DC, blue) and non-DC (grey) metabolites. The panel within the plot illustrates the medians of the two distributions with 95% confidence intervals calculated using bootstrapping (see SI Materials and Methods). (B,C) The LR of enzymes associated with DC and non-DC metabolites (*y*-axis) is plotted for multiple cutoffs defining DC (*x*-axis) in human compared to chimpanzee (B) and rhesus macaque (C).

We found that enzymes that control reactions involving metabolites whose concentrations significantly differ between human and chimpanzee are also more likely to be differentially expressed between the species (Fig. 4a; t-test *P* = 0.065; permutation *P* = 0.017). Though the evidence for a connection between inter-species differences in gene expression levels and the corresponding metabolite concentrations is weak, it is robust. We observed the same pattern regardless of the cutoff used to classify the concentrations of metabolites as different between humans and chimpanzees (Fig. 4b), and when we considered the median difference in estimated expression levels between human and chimpanzee instead of the likelihood ratio statistic (Supplementary Fig. S8). Moreover, enzymes that control reactions involving metabolites whose concentrations significantly differ between human and rhesus macaque are also more likely to be differentially expressed between the species (though this pattern holds only for the 25 metabolites with the strongest evidence for differences in concentration between human and rhesus macaque; Fig. 4c).

**Figure 5:**
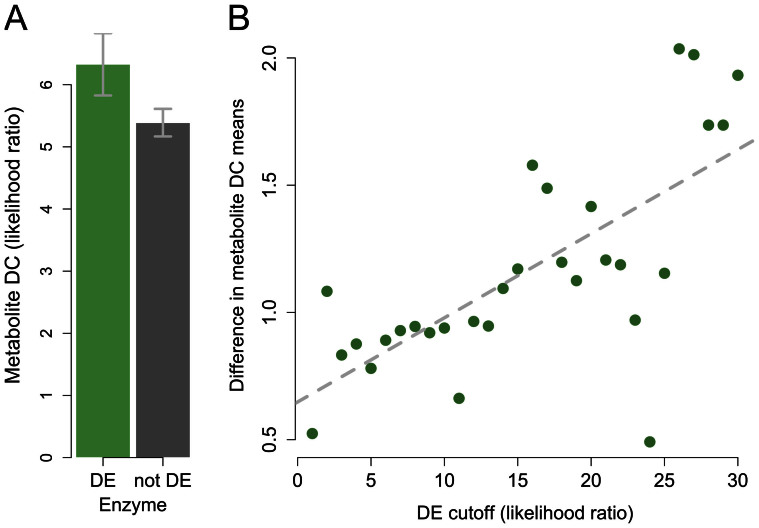
Higher enzyme differential expression between species predicts a higher level of differential concentration for metabolites involved in the same reaction. (A) Mean LR (*y*-axis) for DC between human and chimpanzee in metabolites associated with enzymes that are DE (green) and non-DE (grey). Error bars correspond to 95% confidence intervals calculated using bootstrapping (see SI Materials and Methods). (B) The difference between the means described in A (y-axis) for multiple cutoffs to define enzyme DE (*x*-axis). The dashed line represents a linear regression of the plotted values (correlation significance is P = 1.37 × 10^−5^).

To provide further support for the connection between inter-species gene expression differences and changes in metabolite concentrations we performed an additional analysis in which we stratified the data by the gene expression profiles. Since the correspondence between enzymes and metabolites is not symmetric, this analysis complements the previous result (which is based on stratification of the data by the metabolomics profiles; Fig. 4). Using this complimentary approach we again found that the concentrations of metabolites associated with enzymes that are differentially expressed between human and chimpanzee are more likely to differ between the species (Fig. 5a; permutation *P* = 0.026). This observation is robust with respect to the cutoff used to classify gene expression differences between the species (Fig. 5b). In fact, with more stringent cutoffs to classify inter-species gene expression differences we observed larger differences in the concentrations of the corresponding metabolites. In other words, there is a clear positive correlation between levels of enzyme differential expression and differences in the concentration of associated metabolites (Fig. 5B; correlation *P* < 10^−4^).

## Discussion

We described a comparative analysis of metabolic profiles in the livers of humans, chimpanzees, and rhesus macaques. Our results point to widespread human-specific shifts in liver metabolite concentrations, consistent with recent changes in diet in human evolution. We found that many of the 21 metabolites showing human-specific levels are directly related to documented differences in diets between the species; for example, erythritol, glucose, mono olein, and quinic acid, which have dietary sources unique to humans, and have significantly higher levels in the human liver.

We note that while the natural diets of chimpanzees and rhesus macaques differ, their diets in research colonies, where our samples were collected, are largely similar, which may have intensified the human-specific effect we observed. Moreover, environmental effects that are not diet-related, such as differences in age and sex, may have had an influence on some of the patterns that we discovered as well.

Although sex has been shown to be correlated with differences in gene expression between species^13^, we did not identify a significant sex effect when including sex as a covariate in our model (see supplementary methods). However, we note that the number of individuals from each sex is small, and the study may be underpowered to detect differences between sexes within each species. The gut microbiota, which has been associated with several metabolic phenotypes^24^, presents another dimension of environmental variability that may have an effect on liver metabolism and gene expression. We expect that impact of environmental effects, including diet and the gut microbiome, on metabolic and gene expression profiles, would be the focus of follow-up studies. An especially intriguing direction is the use of computational models that incorporate and assess the contribution of genetic and environmental factors to explaining variability in molecular phenotypes.

The technique used to quantify metabolite concentrations in our study was GC-TOF MS, as this technique yields the largest overview over metabolites smaller than approximately 500 Da, especially the diversity of carbohydrates (mono-, di- and trisaccharides), sugar alcohols, hydroxyl acids (including intermediates of the tricarboxylic acid cycle), amino acids, aromatics, free fatty acids, and ranges of miscellaneous compounds such as purines and pyrimidines. While there is overlap in metabolite coverage with complementary techniques such as liquid chromatography/mass spectrometry, GC-TOF MS is superior in separating isomeric compounds such as fructose and glucose and has better command over data processing software such as mass spectral deconvolution, data processing algorithms^25^ and mass spectral libraries^26^. As every other metabolomic technique, GC-TOF MS is limited in scope; for example, complex lipids such as phosphatidylcholines or thermodegradable metabolites like ATP cannot be analyzed this way. Therefore, this technique may not give a complete picture of the metabolome, and certain metabolites that have an effect on liver function may not be represented in our dataset due to the limitations of the this technology.

Although very little is known about how metabolic profiles differ between species in the liver, Fu et al. have recently performed similar metabolic profiling in the brains of multiple primates, and found a few metabolites with inter-species differences^27^. Considering the 38 metabolites that were included in the analysis by Fu *et al.* as well as the current study, we found that seven have a human-specific pattern in the brain and four in the liver (Supplementary Table S6). Of those, two show a human-specific pattern in both tissues: taurine and oxoproline. However, the directionality of the taurine inter-species pattern in liver and brain is not consistent and the overlap in the two studies is in any case not larger than is expected by chance (Fisher's exact test *P* > 0.3).

Although it is clear that some metabolic differences are due to changes in diet between species, we also found a significant correlation between changes in metabolite concentrations and inter-species differences in the expression of the genes corresponding to the relevant metabolic enzymes in the liver. The observation that enzymes that are differentially expressed are more likely to control reactions that involve differentially concentrated metabolites supports the notion that difference in gene regulation between species likely have a functional impact on downstream differences in metabolic pathways and processes. At this time, however, we cannot distinguish between specific changes in gene regulation between species that are the result of a transient response (e.g., as has been observed in mice fed different diets^28^) from those that result from evolutionary differences in diets. However, we note that among the enzymes that directly interact with diet-related metabolites in Fig. 1, only one enzyme (trehalase, which hydrolyses trehalose) is differentially expressed between species. This supports the notion that the differential expression patterns we observe may not be a transient response to immediate dietary differences.

Moreover, a correlation between inter-primate differences in enzyme expression and metabolite concentration was also observed in the brain^27^. This pattern, which is less likely to be a response to inter-species differences in diet^28^, is similar to the one observed here and thus might indicate that a genetic basis for such differences is likely. In addition, *cis*-regulatory regions of nutrition-related genes show strong evidence for positive selection during human evolution^15^, providing further support for a role for gene regulatory adaptations in facilitating the vast dietary shift in recent human evolution.

In summary, our results suggest an interesting link between human dietary transitions, metabolite concentrations, and gene regulatory processes. Since humans and primates are not experimental models, we cannot distinguish between genetic and purely environmental effects that underlie inter-species differences in metabolite concentrations. Nevertheless, our observations are consistent with previous lines of work suggesting that shifts in diet have had important consequences during human evolution.

## Methods

### Primate samples

Liver samples from non-human primates were collected at necropsy, within four hours of death, by the Yerkes primate center, the Southwest Foundation for Biomedical Research, and MD Anderson Cancer Center. Additional primate tissues were given to us by Anne Stone (Arizona State University). In all cases, non-human primate research was approved by the IACUC at the appropriate institution, and all experiments were performed in accordance with relevant guidelines and regulations. We collected liver tissue samples from adult chimpanzees and rhesus macaques that died of natural causes (such as accidents or fights) or were euthanized due to an illness unrelated to liver. The human adult liver samples were collected for us by the National Disease Research Interchange (NDRI), and by the pathology department at Yale University. The study was exempt from IRB as the human samples were collected post-mortem and de-identified. Detailed information about all samples is available in Table S2. Tissue samples were immediately frozen and maintained at −80C. From each liver sample, we excised three small tissue pieces, each ~100 mg, from different sections of the original, larger sample. This step was conducted on dry ice to avoid thawing the samples.

### Metabolomics experiment

Detailed description of the methods is available in Supplementary Methods. Briefly, eighteen liver samples were collected from adult humans, chimpanzees, and rhesus macaques, with six individuals from each species. 2 mg fresh weight of frozen liver tissues were homogenized using a Retsch ball mill and subsequently extracted with 1 ml of a carefully degassed −20°C cold isopropanol/acetonitrile/water mixture (3:3:2, v/v/v) for 5 min at 4°C. After centrifugation, half of the supernatant was dried and metabolites were derivatized by methoximation and trimethylsilylation as published previously^25^. 0.5 ul of sample was injected into multi-baffled liners and automatic liner exchange for every 10 samples into a Gerstel CIS cold injector at an initial temperature of 50°C (ramped by 12°C sec^−1^ to 250°C) with 25 s splitless time during injection. Chromatography was performed on an Agilent 6890 gas chromatograph with a Restek 30 m × 0.25 mm i.d. × 0.25 um Rtx-5Sil MS column with 10 m integrated guard column at a constant flow of Helium of 1 ml min^−1^ starting at 50°C for 1 min, and then ramped at 20°C min^−1^ to 330°C. Electron ionization mass spectra were acquired at 70 eV from m/z 85–500 at 20 spectra s^−1^ for 20 min run times. Chromatogram acquisition, data handling, automated peak deconvolution, and export of spectra was automatically performed by the Leco ChromaTOF software (v2.32). Data were further processed using the algorithms implemented in the open-source BinBase metabolome database^16^. Metabolites were identified using the Fiehnlib libraries^26^ and missing values were replaced from baseline-subtracted raw data for each individual compound using the BinBase algorithm. The final dataset used here included 399 metabolites detected in all samples, of which 177 had an associated name, and 153 were also found in the KEGG database.

### Statistical analysis

The data were quantile-normalized, and following a principal component analysis we excluded three outlier measurements, and also corrected possible technical artifacts. To identify metabolites that are differentially concentrated between species, the final metabolite data were fitted with a mixed-effects linear model with fixed effect for species and a random effect for individuals. We calculated the ratio of likelihoods of this full model to that of a reduced model, which assumes a similar concentration between each pair of species. We then used this likelihood ratio to estimate a P-value for differential concentration between species (Supplementary Methods). We used expression data that we have previously collected and analyzed for the same samples for which we have metabolomics data^14^. All metabolic pathway information was downloaded from the KEGG database^18^. To identify pathways enriched among metabolites with a human-specific pattern, we used the Fisher's combined P-value across all metabolites in each pathway, as well as the Ingenuity Pathway Analysis tool (http://www.ingenuity.com/).

## Supplementary Material

Supplementary Information Supplementary Information

Supplementary Information Supplementary Table S1
