# Supplementary material for: Comparative metabolomics in primates reveals the effects of diet and gene regulatory variation on metabolic divergence
Source: Sci Rep. 2014 Jul 28;4:5809. doi: 10.1038/srep05809 (PMC4894427; doi:10.1038/srep05809)
Supplement: Supplementary Information [file srep05809-s1.pdf]

Comparative metabolomics in primates reveals the effects of diet and gene regulatory variation on metabolic divergence

Ran Blekhman[1,2,*], George H. Perry[3], Sevini Shahbaz[4], Oliver Fiehn[4], Andrew G. Clark[5], Yoav Gilad[6,*]

1. Department of Genetics, Cell Biology, and Development, University of Minnesota, Minneapolis, MN, 55455
2. Department of Ecology, Evolution, and Behavior, University of Minnesota, St. Paul, MN, 55108
3. Department of Biology, Penn State University, University Park, PA, 16802
4. UC Davis Genome Center, University of California, Davis, CA, 95616
5. Department of Molecular Biology and Genetics, Cornell University, Ithaca, NY, 14853
6. Department of Human Genetics, University of Chicago, Chicago, IL, 61637

* Corresponding Authors: Ran Blekhman (blekhman@umn.edu), Yoav Gilad (gilad@uchicago.edu)

Supplementary Information

Includes Supplementary Methods, Supplementary Tables S2-S6, and

Supplementary Figures S1-S10

Supplementary Methods

Sample preparation

For this analysis we used 18 liver samples from three primate species (human, chimpanzee, and rhesus macaque), with 6 individuals from each species. Liver samples from non-human primates were collected at necropsy, within four hours of death, by the Yerkes primate center, the Southwest Foundation for Biomedical Research, and MD Anderson Cancer Center. Additional primate tissues were given to us by Anne Stone. In all cases, we collected liver tissue samples from adult chimpanzees and rhesus macaques that died of natural causes (such as accidents or fights) or were euthanized due to an illness unrelated to liver. The human adult liver samples were collected for us by the National Disease Research Interchange (NDRI), and by the pathology department at Yale University (with IRB approval). Detailed information about all samples is available in Table S2.

Tissue samples were immediately frozen and maintained at -80C. From each liver sample, we excised three small tissue pieces, each ~100 mg, from different sections of the original, larger sample. This step was conducted on dry ice to avoid thawing the samples.

Metabolomics methodology

To quantify metabolite levels in liver samples, we have applied gas chromatography/time-of-flight mass spectrometry (GC-TOF MS), as this technique yields the largest overview over metabolites smaller than approximately 500 Da, especially the diversity of carbohydrates (mono-, di- and trisaccharides), sugar alcohols, hydroxyl acids (including intermediates of the tricarboxylic acid cycle), amino acids, aromatics, free fatty

acids, and ranges of miscellaneous compounds such as purines and pyrimidines. While there is overlap in metabolite coverage with complementary techniques such as liquid chromatography/mass spectrometry, GC-TOF MS is superior in separating isomeric compounds such as fructose and glucose and has better command over data processing software such as mass spectral deconvolution, data processing algorithms [1] and mass spectral libraries [2]. As every other metabolomic technique, GC-TOF MS is limited in scope; for example, complex lipids such as phosphatidylcholines or thermodegradable metabolites like ATP cannot be analyzed this way.

Metabolomics data acquisition

2 mg liquid-nitrogen frozen liver tissue samples were homogenized at 25 Hz with 3 mm i.d. steel balls for 30 s and immediately placed back into liquid nitrogen, afterwards. Samples were taken out one by one and 1 ml of a carefully degassed -20°C cold isopropanol/acetonitrile/water mixture (3:3:2, v/v/v) were added and shaken for 5 min at 4°C to extract metabolites and simultaneously precipitate proteins. After centrifugation at 12,800 x *g* for 2 min, 90% of the supernatant was removed, separated into two equal aliquots, transferred to a 1.5 ml Eppendorf tube and concentrated to dryness in a vacuum concentrator. Samples were cleaned up by adding 500 ul of a degassed acetonitrile/water mixture (1/1, v/v), vortexing, centrifugation, decanting the supernatant and drying in a speed vac concentrator. This step removes triglycerides and most of the complex lipids, but not phytosterols or free fatty acids and is needed because otherwise, the involatile matrix lipids would interfere with the derivatization reaction of primary amines and amino acids.

C08-C30 fatty acid methyl esters in chloroform were added as internal retention index (RI) markers. Subsequently, metabolites were derivatized by adding 10 ul of a solution of 20 mg/ml of 98% pure methoxyamine hydrochloride (CAS number 593–56-6, Sigma-Aldrich) in pyridine for 90 min at 28°C. Afterwards 90 ul of N-methyl-N-trimethylsilyltrifluoroacetamide (MSTFA, Sigma-Aldrich) was added for trimethylsilylation of acidic protons and shaken at 37°C for 30 min. The reaction mixture was transferred to a 2 ml clear glass auto-sampler vial with micro-insert (Agilent) and closed using a 11 mm T/S/T crimp cap (MicroLiter). A Gerstel automatic liner exchange system with a MPS2 dual rail multi-purpose sampler was used in conjunction with a Gerstel CIS cold injection system. For every 10 samples, a fresh multi-baffled liner was inserted (Gerstel #011711-010-00). Before and after each injection, the 10 ul injection syringe was washed three times with 10 ul ethyl acetate. 1 ul sample was filled using 39 mm vial penetration at 1ul sec-1 filling speed, injecting 0.5 ul at a 10 ul s-1 injection speed at an initial temperature of 50°C which was ramped by 12°C sec-1 to a final temperature of 250°C and held for 3 min. The injector was operated in split-less mode, opening the split vent after 25 sec. Samples were injected between 2–24 h after derivatization using randomized sequences controlled by the laboratory information management and database system. An Agilent 6890 gas chromatograph was used with a 30 m long, 0.25 mm internal diameter Rtx-5Sil MS column with 0.25 lm 95% dimethyl/5%diphenyl polysiloxane film and an additional 10 m integrated guard column was used (Restek). Separation was achieved by 99.9999% pure helium with built-in purifier (Airgas) at a constant flow of 1 ml min-1. The oven temperature was held constant at 50°C for 1 min, and then ramped at 20°C min-1 to 330°C, and held constant for 5 min. The transfer line

temperature was set to 280 °C. After 290 s solvent delay, filament 1 was turned on at an ion source temperature of 250 °C. Electron ionization mass spectra were acquired at 70 eV with mass resolving power R = 600 from m/z 85-500 at 20 spectra s-1 and 1850 V detector voltage without turning on the mass defect option. Recording ended after 1200 s. The instrument performed auto-tuning for mass calibration using FC43 (perfluorotributyl-amine) before starting analysis sequences.

Metabolomics data processing

Chromatogram acquisition, data handling, automated peak deconvolution, and export of spectra was automatically performed by the Leco ChromaTOF software (v2.32). Peak picking was achieved in ChromaTOF (v2.32) at signal/noise levels of 10:1 throughout the chromatogram with baseline subtraction just above the noise level, no smoothing, 3 s default peak widths, automatic mass spectral deconvolution and peak detection and export of result spectra as *.csv files in addition to export of raw data in open-access *.cdf formats. Data were further processed using the algorithms implemented in the open-source BinBase metabolome database [3]. This algorithm used the settings: validity of chromatogram (<10 peaks with intensity >10^7 counts sec-1), unbiased retention index marker detection (MS similarity >800 and exceeding thresholds for ion ratio abundances for high m/z marker ions), retention index calculation by 5th order polynomial regression. Spectra were cut to 5% base peak abundance, and matched to database entries from most- to least-abundant spectra using the following matching filters: retention index window ±2000 units (equivalent to about ±2 sec retention time), validation of unique ions and apex masses (unique ion must be included in apex masses

and present at >3% of base peak abundance), mass spectrum similarity that must fit criteria dependent on peak purity and signal/noise ratios, optional ion ratio settings to distinguish peaks with high similarity, and a final isomer filter (annotating the isomer spectrum with the closest RI fit). Novel spectra not yet included in BinBase were automatically entered as new database entries if their signal-to-noise ratio >25, purity <1.0 and presence in the biological study design class was >80%. This filter ensured that (i) signals were reported that had never been detected previously in any other sample, but (ii) only signals were reported that can be assumed to be biologically relevant using relatively abundant and pure signals and ensuring that these are positively detected in most of the biological replicates. Signal intensities were reported as peak heights using the unique ion as default, unless an alternative quantification ion was manually set in the BinBase administration software Bellerophon. All known artifact peaks such as internal standards, column bleed, plasticizers or reagent peaks were assigned by BinBase but not exported for further statistical calculations. Metabolites were identified using the Fiehnlib libraries consisting of over 1,200 authentic compounds and referenced using PubChem identifiers [2]. Daily quality controls were used comprising method blanks and five-point calibration curve samples of 31 pure reference compounds which were repeatedly analyzed over the full analytical sequence in addition to injection of one QC sample for every 10 biological samples. A quantification report table was produced for all database entries that were positively detected in more than 50% of the samples of a study design class. This procedure results in 10–30% missing values, which could be caused by true negatives (compounds that were below the detection limit in a specific sample) or false negatives (compounds that were present in a specific sample but that did not match

quality criteria in the BinBase algorithm). A subsequent post-processing module was employed to automatically replace missing values from the *.cdf files with the following parameters: for each positively detected metabolite, the average retention time was calculated for the day of analysis. Subsequently, for each chromatogram and each missing value, the intensity of the quantification ion at this retention time was extracted by seeking its maximum value in a retention time region of ± 1 s and subtracting the minimum (local background) intensity in a retention time region of ±5 s around the peak maximum. The resulting report table therefore did not have any missing values.

Metabolomics data normalization and statistics

Result files were normalized by calculating the sum intensities of all structurally identified compounds for each sample (i.e. those signals that had been positively identified in the data pre-processing schema outlined above), and subsequently dividing all data associated with a sample by the corresponding metabolite sum. The resulting data were multiplied by the average sum of all identified metabolites detected in the study (total average metabolome transformation), disregarding unknown metabolites as these might potentially also comprise artifacts. Intensities of identified metabolites with more than one peak (e.g. for the syn- and anti-forms of methoximated reducing sugars, or amino acids with different derivatization status of amine groups) were summed to only one value in the transformed data set. The original non-transformed data set was retained for retrospective analysis. When comparing classes of samples with biologically different sum concentrations of identified metabolites ($p<0.05$), these class averages were used for mean transformations. The final metabolomics data consists of three replicates for each

sample, for a total of 54 (3*6*3) measurements for each metabolite, and is available as a separate data file (Dataset S1). The dataset included information for 399 metabolites detected in all samples, of which 177 had an associated name, and 153 were also found in the KEGG database.

Expression data

We used expression data that we previously collected and analyzed [4]. Briefly, the data was collected using a multi-species microarray, containing orthologous probes from three primate species: human, chimpanzee, and rhesus macaque. The array contains probes for 18,109 genes (368,678 probes in total). The data includes gene expression estimates from six individuals from three tissues (liver, kidney cortex and heart muscle), from each of the three species. Complete information on sample collection, study design, array hybridizations, low-level analysis, and quality control is available in Blekhman et al. (2008).

Low-level analysis and quality control of the metabolite data

First, we normalized the metabolite data using the quantile normalization approach, with the normalize.quantiles function in the R library preprocessCore version 1.8. To assess to quality of the data, we performed principal component (PC) analysis on the normalized data, using the princomp function in R. and plotted the first vs second principal components (figure S1C). We note that the PCs separate the samples based on the batch (replicate) in which it was run, with PC1 splitting the first batch from the rest, and PC2 separating the second and third batches. Since this variation is technical, and

could lead to spurious results, we decided to remove this effect and correct for it before moving on with the analysis. We also note that PC3 does not correspond to any expected biological division of data, while PC4 separates the samples based on their species (see figure S1D). Since the species effect is expected to be the main factor explaining the variance between samples, we concluded that PC3 is a technical effect, and decided to correct for this unexplained effect as well.

To do so, we analyzed the normalized data with a metabolite-wise linear model including the replicate and third PC as factors. We generated the corrected data by summing the intercept and residuals for each metabolite, thus regressing out the model factors, namely the batch and the third PC.

In addition to correcting for non-biological variance, we also used the PC analysis, together with a heatmap plot of sample pairwise correlations, to identify outliers in the metabolite data. We found three outlying samples that did not cluster with their other two replicates: (1) human sample 56720 replicate 2 (can be clearly seen in figure S1C), (2) human sample 56655 replicate 3, and (3) rhesus macaque sample YN05-349 replicate 3. After excluding these samples from the data, we repeated the PC analysis, and found no visible confounding effect and outliers (figure S1A and S1C). Moreover, we see the species as the first and second PCs. In addition, a heatmap plot of the normalized, corrected, outlier-excluded data shows a clear separation (figure S2).

Metabolic pathway data

All metabolic pathway information was downloaded from KEGG [5] via the ftp site at http://www.genome.jp/kegg/download on February 2011. The tables that were

downloaded are ‘rpair’, ‘reaction’, ‘enzyme’, and ‘compound’. Of the 177 metabolites with known name in our dataset, 153 also had a KEGG ID. We extracted from the ‘compound’ table information for these metabolites, including the pathways they each metabolite is included in, and the names of enzymes associated with each metabolite. To get information on pairs of metabolites involved in the same reaction, as well as the KEGG enzyme controlling each reaction, we used the ‘rpair’ table. We found 133 such metabolite “pairs” where both metabolites are included in our dataset.

We then matched the gene symbols of the genes represented on our expression array to the KEGG enzyme names, and found a KEGG enzyme record for 2,626 of the 17,231 genes. We found 1043 unique enzyme-metabolite associations in KEGG where we have both the metabolite concentration level and enzyme expression level.

Identifying differences in metabolite concentrations between species

To identify metabolites with differences in concentrations between species, we applied the following linear model on the levels of each metabolite:

$$y_{sij} = \mu_s + \alpha_{si} + \varepsilon_{sij}$$

where y_{si} is the normalized log concentration level from species s in individual i and replicate j, μ_s is the expected log concentration level in species s, α_i is a random effect to capture the variance between individuals within each species, and ε_{si} is the error term.

To identify metabolites differentially concentrated (DC) between species we used a set of three likelihood ratio tests, each comparing the likelihood of the full model above with that of a reduced model, which assumes there is a similar concentration level across the species. We calculated a likelihood ratio (LR) statistic for each pairwise comparison,

and estimated a P-value under the assumption that the LR has a chi-square distribution with one degree of freedom. We then calculated the false discovery rate associated with these P-values using the approach of [6]. Finally, we used a threshold of q-value < 0.05 and identified 122, 96, and 29 metabolites with different concentrations between human and chimpanzee, human and rhesus macaque, and chimpanzee and rhesus macaque, respectively (see Figure S3). Given the small number of differences between chimpanzee and rhesus macaque, we focused most of the following analyses on the human-chimpanzee and human-rhesus macaque comparisons, which were more informative.

Next, we aimed to find metabolites with a human-specific concentration level, i.e., metabolites that show a significantly higher (or lower) level in human, but a similar level in the two non-human species. To do so, we combined the results of the three pairwise tests described above, and picked genes with a $P<0.01$ for human-chimpanzee and human-rhesus macaque comparison, and $P>0.1$ for chimpanzee-rhesus macaque. We identified 35 metabolites with the expected pattern, of which 21 have a known name (shown in Figure 1).

Human-specific metabolic concentrations in pathways

We then wanted to identify KEGG pathways that show evidence for human-specific metabolic concentrations. To do so, for each pathway in KEGG we combined the pairwise P-values for differential concentration over all the metabolites in the pathway using Fisher's method, and considered pathways with a combined, Bonferroni-corrected $P<0.05$ for the human-chimpanzee and human-rhesus macaque differences, and combined $P>0.1$ for the chimpanzee-rhesus difference. Table S3 lists the most enriched pathways,

including the metabolites included in the analysis in each pathway, and the combined pairwise P-values.

Identifying differentially expressed genes

For all subsequent analyses we excluded probes that did not have corresponding orthologs in all three species (i.e., we only consider probes that have the human, chimpanzee, and rhesus macaque species-specific versions on the array – we refer to these as the "corresponding orthologous probes"). Following this step, we excluded genes that were represented by fewer than three corresponding orthologous probes across all species. Thus, the total number of genes included in all subsequent analyses was 17,231 (95% of genes originally included on the array). Expression estimates were obtained from [4].

To identify genes that are differentially expressed (DE) between human and chimpanzee within a tissue, we used likelihood ratio (LR) tests within the frame work of nested mixed linear models, as previously described [4]. Briefly, we estimated the maximum likelihood of the full model as well as that of a reduced (null) model, in which we assume that the expression level in human and chimpanzee is similar. We then calculated $-2\cdot$(log-likelihood ratio) between the fits of the reduced and full models. We expect genes that deviate from the null (i.e., genes that are truly differentially expressed between human and chimpanzee) to have higher values of this statistic.

Correlation between the number of reactions and differential concentration

In order to test correlations among the number of metabolic reactions a metabolite is involved in (commonly called "connectivity") and the level of DC between species, we first examined the distribution of the number of reactions per metabolite extracted from KEGG. As can be seen in Figure S4, the distribution is enriched with low-reaction metabolites, but has a long tail of high-connectivity metabolites. We show results using a cutoff of 20 reactions to distinguish the two groups, but results remain unchanged for multiple other cutoff choices. After splitting the metabolites to low- and high-connectivity compounds, we plotted the distribution of likelihood ratios for differential concentration between species pairs in Figure S5.

Permutation test for difference in medians

We used a permutation test to estimate the significance of observed differences between the medians of values for different groupings of metabolites (e.g. likelihood ratio for metabolites involved in reactions controlled by differentially expressed vs. non-differentially expressed enzymes). For this purpose, we define the difference between the medians (or means) of the two groups as D. Then, we randomly divided all the values into two groups of same sizes as observed, and calculated the medians of the random groups. This permutation was repeated 10,000 times, and each time the difference between the medians of the two randomly selected groups (D_i) was recorded. The test P-value was defined as the number of times where $D_i \geq D$, divided by 10,000.

References

1. Fiehn O, Wohlgemuth G, Scholz M, Kind T, Lee do Y, et al. (2008) Quality control for plant metabolomics: reporting MSI-compliant studies. Plant J 53: 691-704.
2. Kind T, Wohlgemuth G, Lee do Y, Lu Y, Palazoglu M, et al. (2009) FiehnLib: mass spectral and retention index libraries for metabolomics based on quadrupole and time-of-flight gas chromatography/mass spectrometry. Anal Chem 81: 10038-10048.
3. Fiehn O, Wohlgemuth G, Scholz M (2005) Setup and annotation of metabolomic experiments by integrating biological and mass spectrometric metadata. Proc Lect Notes Bioinformatics 3615: 224-239.
4. Blekhman R, Oshlack A, Chabot AE, Smyth GK, Gilad Y (2008) Gene Regulation in Primates Evolves under Tissue-Specific Selection Pressures. PLoS Genet 4: e1000271.
5. Kanehisa M, Goto S, Sato Y, Furumichi M, Tanabe M (2012) KEGG for integration and interpretation of large-scale molecular data sets. Nucleic Acids Res 40: D109-114.
6. Storey JD, Tibshirani R (2003) Statistical significance for genomewide studies. Proc Natl Acad Sci U S A 100: 9440-9445.
7. Fu X, Giavalisco P, Liu X, Catchpole G, Fu N, et al. (2011) Rapid metabolic evolution in human prefrontal cortex. Proc Natl Acad Sci U S A 108: 6181-6186.

Supplementary Tables

Table S1 contains the metabolite concentration levels used in this study, and is provided in a separate data file.

Table S2. Information on the liver samples used in this study.

Sample	Source	Species	ID	sex
H1	Yale	Human	Yale 1	m
H2	Yale	Human	Yale 2	m
H3	Yale	Human	Yale 3	m
H4	Yale	Human	Yale 4	m
H5	NDRI	Human	56655	f
H6	NDRI	Human	56720	f
C1	Yerkes	Chimpanzee	YN05-400, Jeanie	f
C2	Yerkes	Chimpanzee	YN06-108, Beleka	f
C3	MD Anderson	Chimpanzee	MDANDER	m
C4	Yerkes	Chimpanzee	YN06-147, Iyk	m
C5	Yerkes	Chimpanzee	c0547, Keith	m
C6	A.Stone	Chimpanzee	YN95-427	m
R1	Yerkes	Rhesus macaque	YN05-349, RFj9	f
R2	Yerkes	Rhesus macaque	YN05-82, RFh3	m
R3	A.Stone	Rhesus macaque	13330-305, R1333	m
R4	A.Stone	Rhesus macaque	19935-305, R1999	m
R5	Yerkes	Rhesus macaque	YN04-311	f
R6	Yerkes	Rhesus macaque	17602	m

Table S3. Pathways over-represented with metabolites showing a human-specific pattern.

Pathway Name	Metabolites	P_{H-C}	P_{H-R}	P_{C-R}
**Primary bile acid biosynthesis**	taurine, glycine, cholesterol	8.60E-05	3.34E-05	0.983767682
**Lysine degradation**	lysine, glycine, 2-aminoadipic acid, glutaric acid, 5-aminovaleric acid	0.000108626	2.12E-05	0.949036076
**Arginine and proline metabolism**	urea, proline, glutamine, fumaric acid, creatinine, aspartic acid, ...	5.36E-05	0.003132915	0.635359913
**Phenylalanine metabolism**	phenylalanine, fumaric acid, tyrosine, succinic acid, salicylic acid, ...	4.44E-05	0.001355468	0.956585677
**Phenylalanine, tyrosine and tryptophan biosynthesis**	phenylalanine, tyrosine, tryptophan, quinic acid	0.006070129	0.007642205	0.968361491
**Taurine and hypotaurine metabolism**	cysteine, alanine, taurine	0.003953963	6.41E-05	0.416250497
**Cyanoamino acid metabolism**	serine, aspartic acid, tyrosine, glycine, asparagine, cyano-L-alanine	0.000499792	0.001509633	0.958270424
**Glutathione metabolism**	oxoproline, cysteine, glycine, glutamic acid, dehydroascorbate, ...	2.50E-07	5.42E-07	0.909925842
**Naphthalene degradation**	salicylic acid, salicylaldehyde	0.002293026	0.006249661	0.946095694
**Thiamine metabolism**	cysteine, tyrosine, glycine	0.004317159	0.009294191	0.916033897
**Tropane, piperidine and pyridine alkaloid biosynthesis**	phenylalanine, lysine, isoleucine, putrescine	0.000114158	0.001362611	0.927163623
**Metabolic pathways**	xanthine, valine, uric acid, urea, uracil, threonine, serine, proline, ...	6.98E-18	2.92E-14	0.936975057
**Biosynthesis of secondary metabolites**	xanthine, valine, threonine, serine, proline, phenylalanine, methionine, ...	1.94E-10	6.18E-06	0.994914412
**ABC transporters**	valine, urea, threonine, serine, proline, phenylalanine, lysine, isoleucine, ...	1.49E-05	2.30E-05	0.994763357
**Protein digestion and absorption**	valine, threonine, serine, proline, phenylalanine, methionine, lysine, ...	0.000276889	6.70E-05	0.979072152
**Bile secretion**	uric acid, glucose, cholesterol, salicylic acid, spermidine, ...	0.001121257	0.005314515	0.998908375
**Biosynthesis of phenylpropanoids**	phenylalanine, fumaric acid, 3-phosphoglycerate, citric acid, ...	0.000786858	0.000757432	0.981246965

Table S4. Cellular and molecular functions in the Ingenuity database enriched among the metabolites shown in Figure 2 (top 5 categories are shown).

Functional Category	P-value	Metabolites
**Cell-To-Cell Signaling and Interaction**	3.94E-02	D-glucose,glycine
**Energy Production**	3.66E-02	D-glucose,glycine,citric acid
**Skeletal and Muscular System Development and Function**	3.97E-02	putrescine,D-glucose,glycine,taurine
**Cell Morphology**	4.9E-02	AMP,D-glucose,glycine,taurine
**Cellular Function and Maintenance**	4.9E-02	AMP,D-glucose,salicylic acid,glycine,taurine

Table S5. Canonical pathways in the Ingenuity database enriched among the metabolites shown in Figure 2 (top 5 categories are shown).

Functional Category	P-value	Metabolites
**Lysine Degradation**	1.10E-03	glutaric acid,2-aminoadipic acid,glycine
**Insulin Receptor Signaling**	1.35E-03	AMP,D-glucose
**Nitrogen Metabolism**	1.82E-03	AMP,glycine,taurine
**Glutathione Metabolism**	1.02E-02	pyrrolidonecarboxylic acid,glycine
**Cyanoamino Acid Metabolism**	1.29E-02	glycine,3-cyano-L-alanine

Table S6. Metabolites found in Fu et al. [7] and also included in our analysis.

Metabolite[1]	Hspec.CBC[2]	Hspec.PFC[3]	inLiver[4]	Hspec.liver[5]
3-hydroxybutyric acid	0	0	0	0
glycerol	0	0	1	0
leucine	0	0	1	0
isoleucine	0	0	1	0
benzoic acid	0	0	1	0
serine	0	0	1	0
succinic acid	0	0	1	0
threonine	0	0	1	0
fumaric acid	0	0	1	0
erythronic acid	0	0	0	0
4-hydroxyproline	0	0	1	0
gaba	0	0	0	0
threonic acid	0	0	1	0
methionine	0	0	1	0
creatinine	0	0	1	0
oxoproline	0	1	1	1
glutamate	0	1	0	0
nicotinamide	0	0	1	0
putrescine	0	0	1	1
dodecanoic acid	0	0	0	0
glutamine	0	0	1	0
phenylalanine	0	0	1	0
glucose-1-phosphate	0	0	1	0
ornithine	0	0	1	0
aspartic acid	0	0	1	0
glycerol-3-phosphate	0	0	0	0
glucose	0	0	1	1
citricacid	0	0	0	0
dehydroascorbic acid	0	0	0	0
histidine	0	1	1	0
pantothenic acid	0	0	1	0
hexadecanoic acid	0	1	0	0
octadecenoic acid	0	1	0	0
6-phosphogluconic acid	0	0	0	0
tryptophan	0	0	1	0
uridine	0	0	1	0
alanine	0	0	1	0
butyricacid	0	0	0	0
tetradecanoic acid	0	0	0	0
cholesterol	0	0	1	0

hydroxypyridine	0	1	0	0
3-hydroxypyridine	0	0	0	0
ribitol	0	1	1	0
fructose	0	0	1	0
glycerol-2-phosphate	0	0	0	0
tyrosine	0	0	1	0
pyruvicacid	0	0	0	0
heptadecanoic acid	0	0	1	0
valine	0	0	1	0
oxalic acid	0	0	1	0
decanoic acid	0	0	0	0
taurine	1	0	1	1
tetracosanoic acid	0	1	0	0
glycolic acid	0	1	1	0
2-amino adipic acid	1	0	0	0
fructose2	0	0	0	0
spermidine	0	1	1	0
glucose-6-phosphate2	0	0	0	0
ascorbic acid	1	0	1	0
adipic acid	0	0	1	0

[1] Metabolite name, listing all metabolites with a known name from Fu et al.
[2] Whether the metabolite has a human-specific pattern in the CBC (Fu et al.).
[3] Whether the metabolite has a human-specific pattern in the PFC. (Fu et al.)
[4] Whether the metabolite was identified and included in our analysis.
[5] Whether we found the metabolite to have a human-specific pattern in the liver.

Supplementary Figures

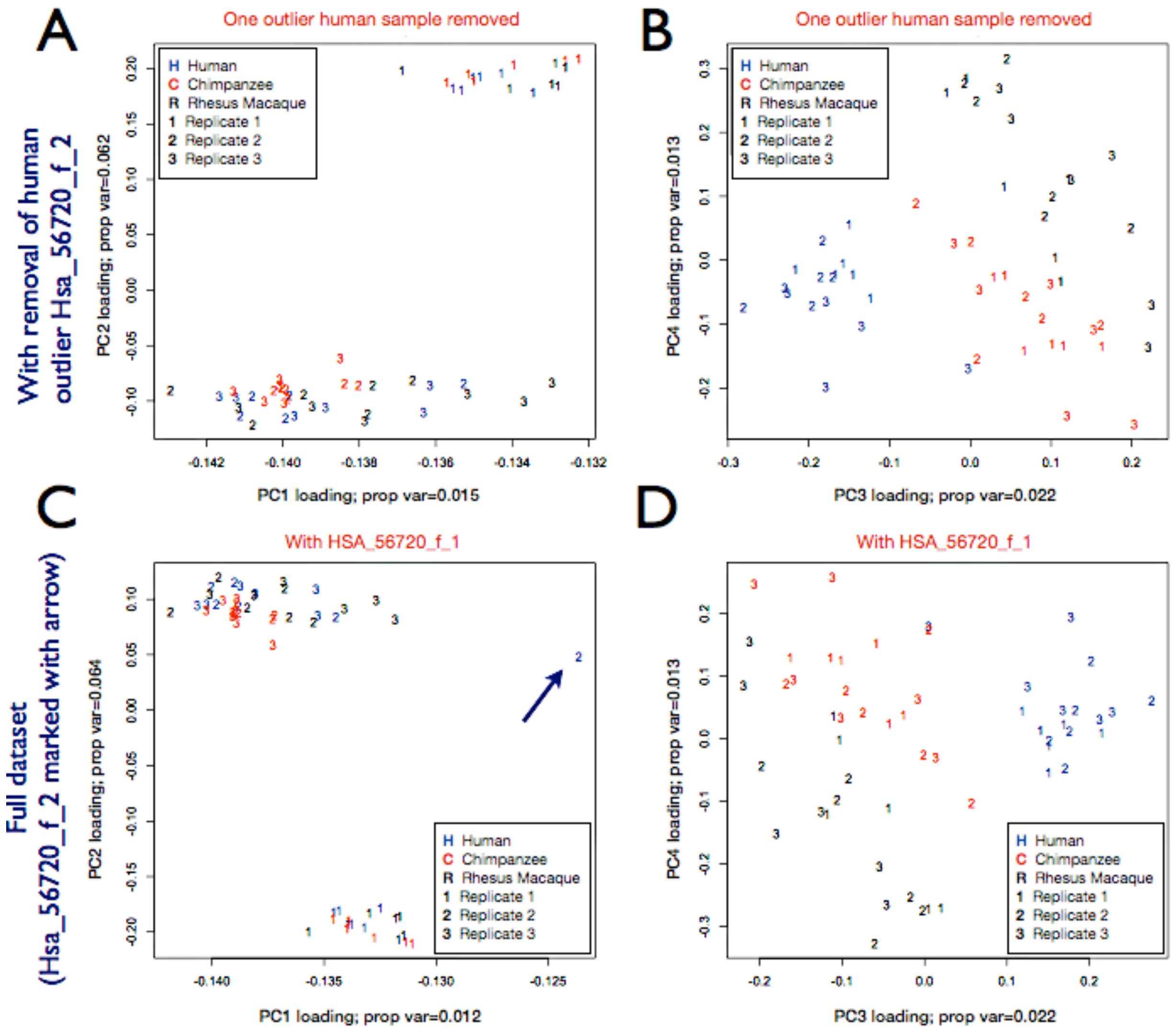

Figure S1. Principal component (PC) analysis of the normalized metabolite data, showing the first vs. second PC (A,C) and third vs. fourth PC (B,D) on the x and y axes, respectively. (A) Data after removal of outlier sample (HSA_56720_f_2); (B) data after removal of outlier and following correction for batch effect; (C) full data, before removal of outlier, PC1 vs. PC2; (D) full data, before removal of outlier, PC3 vs. PC4. Numbers correspond to the three batches, and colors correspond to the species (blue-human, red-chimpanzee, black-rhesus macaque).

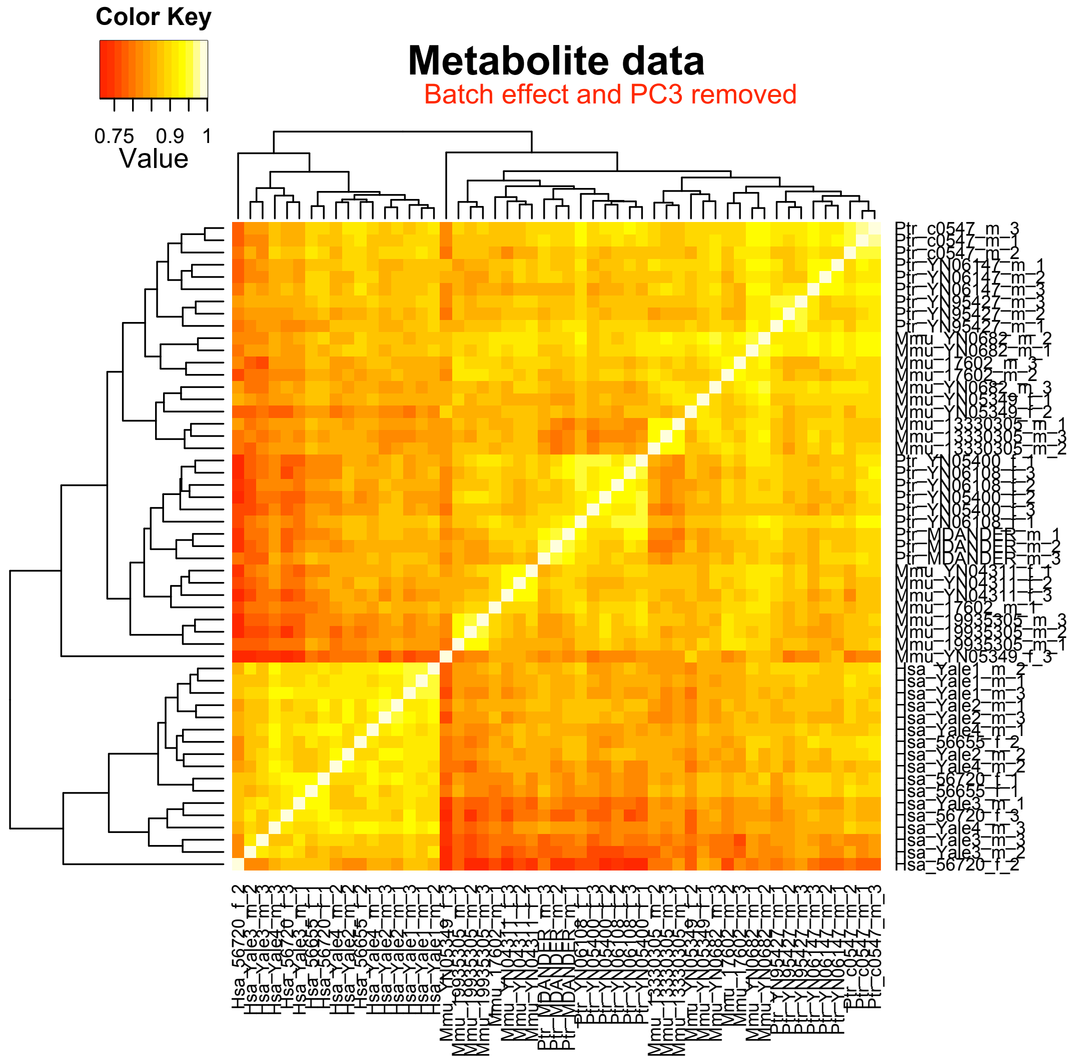

Figure S2. Heatmap of pairwise (Spearman) correlations of normalized and corrected metabolite levels between all samples. Colors represent different R^2 values as indicated in the color key at the top, with lighter colors indicate higher correlation. The dendrogram, which was used to order the rows and columns of the heatmap, which is shown at the top and left sides, was generated by clustering the pairwise correlation matrix using a Euclidean distance metric and complete agglomeration.

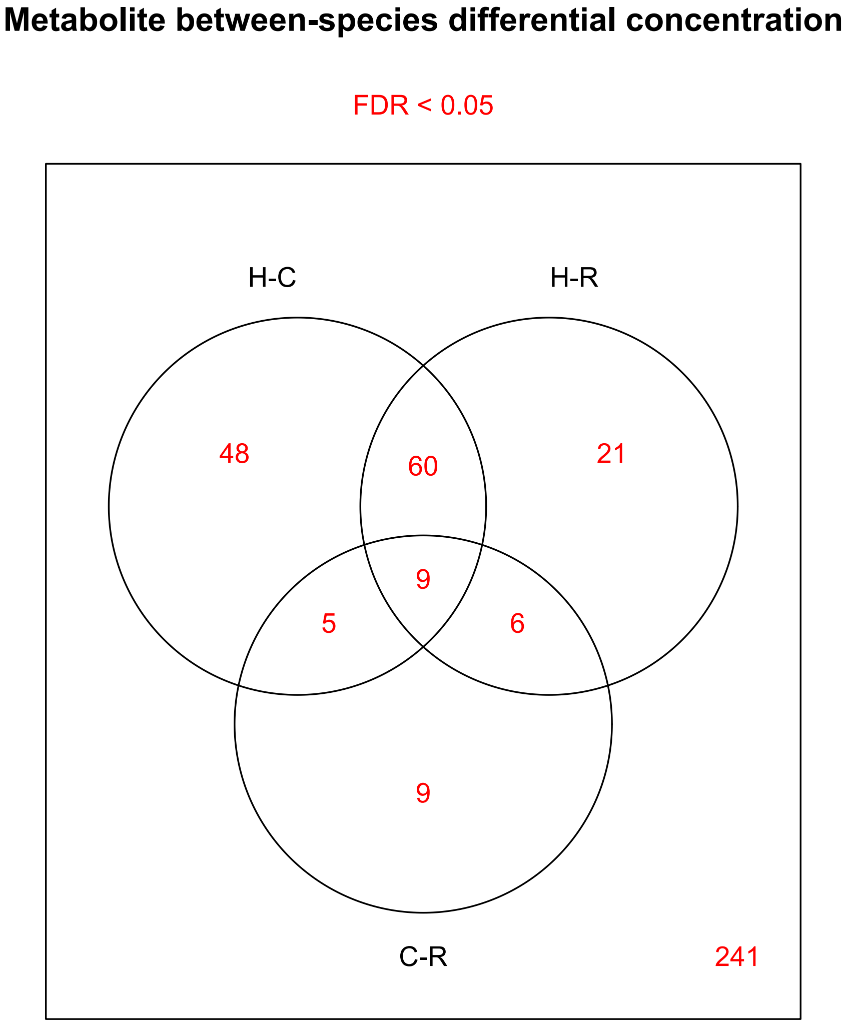

Figure S3. Proportion of DC metabolites between each pair of species (H: human, C: chimpanzee, R: rhesus macaque).

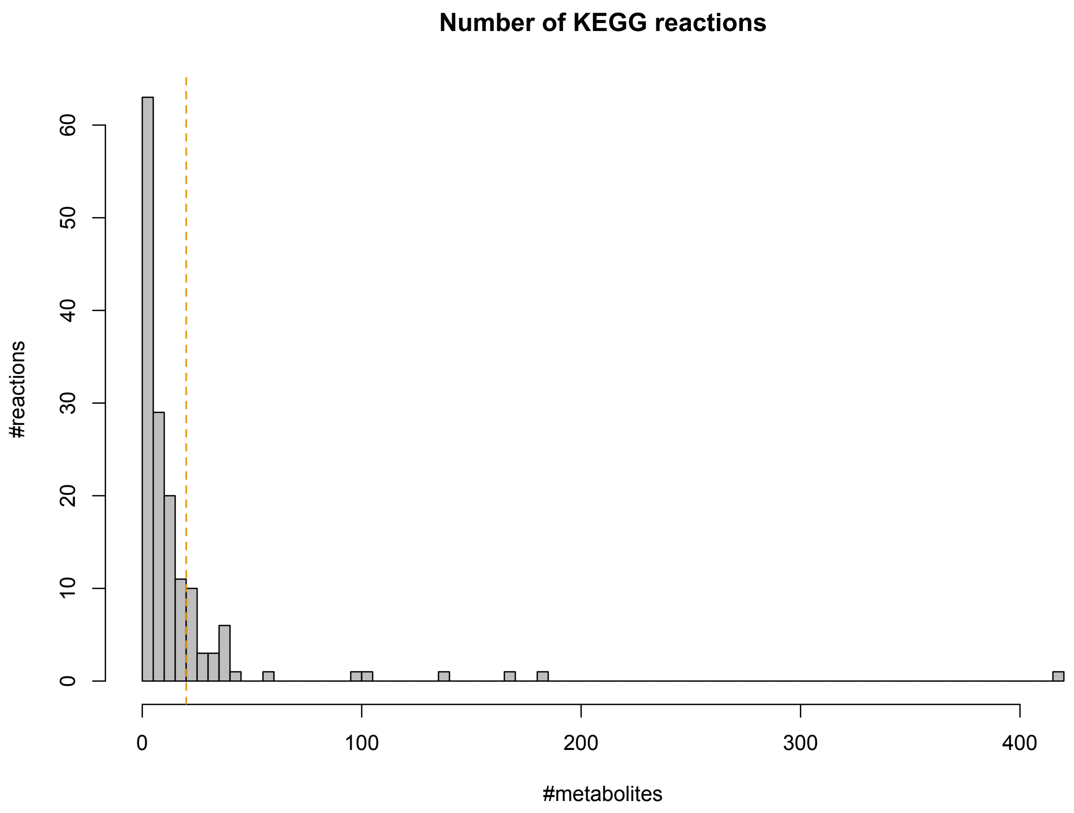

Figure S4. Histogram showing the distribution of number of reactions per metabolite, using all the metabolites in our dataset for which we found a record in KEGG. The orange horizontal line shows the selected cutoff for defining high- and low-reaction metabolites.

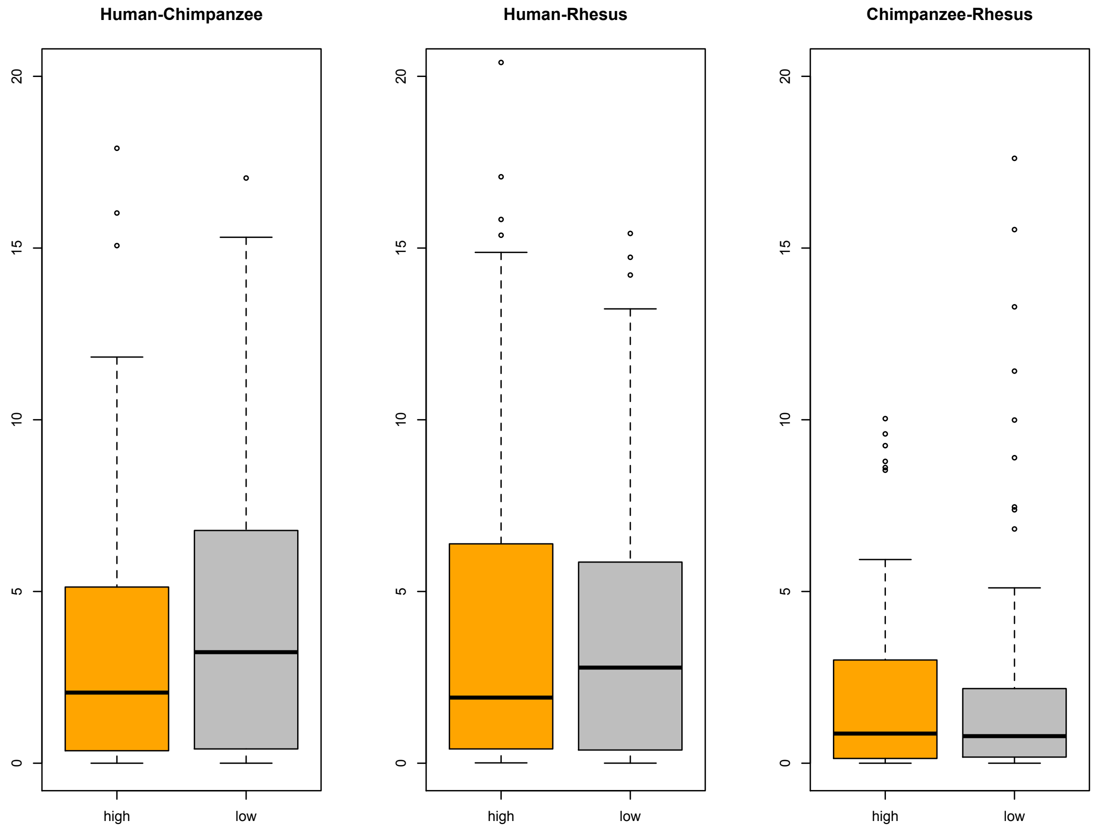

Figure S5. Distribution of likelihood ratios for differential concentration between pairs of species, broken down into high-reaction metabolites (orange) and low-reaction metabolites (grey).

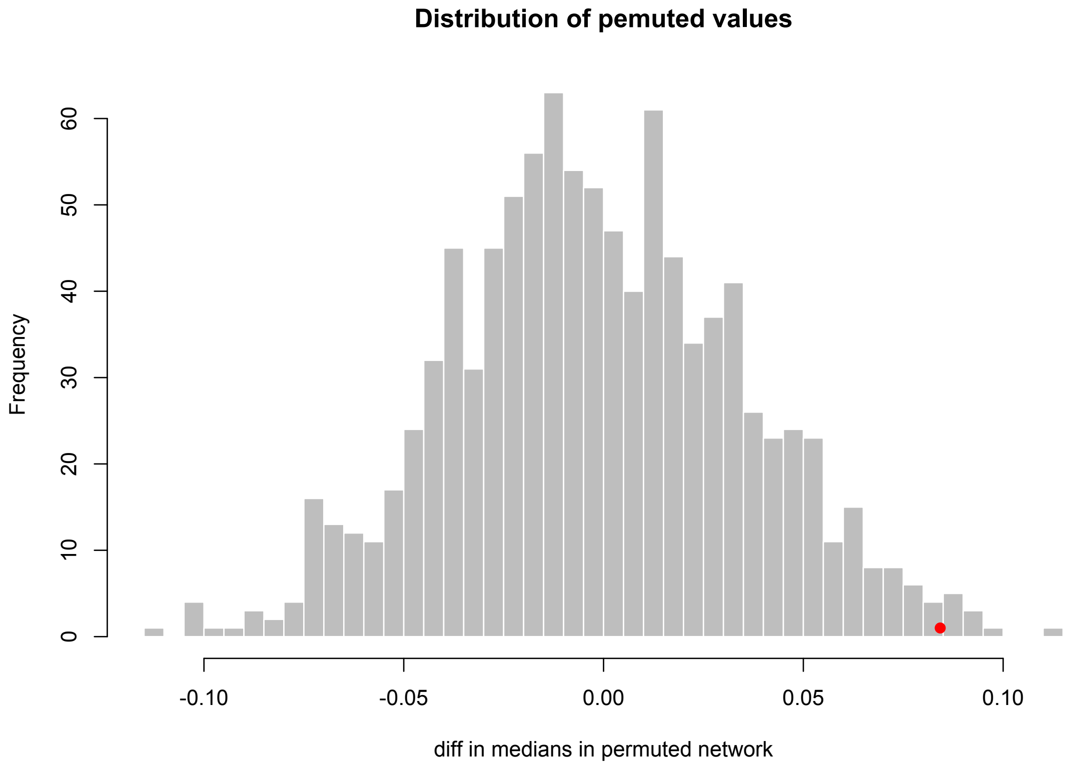

Figure S6. The distribution of difference in medians between paired and unpaired metabolites calculated from 1000 permutations of the real network. The red dot marks the observed unpermuted value.

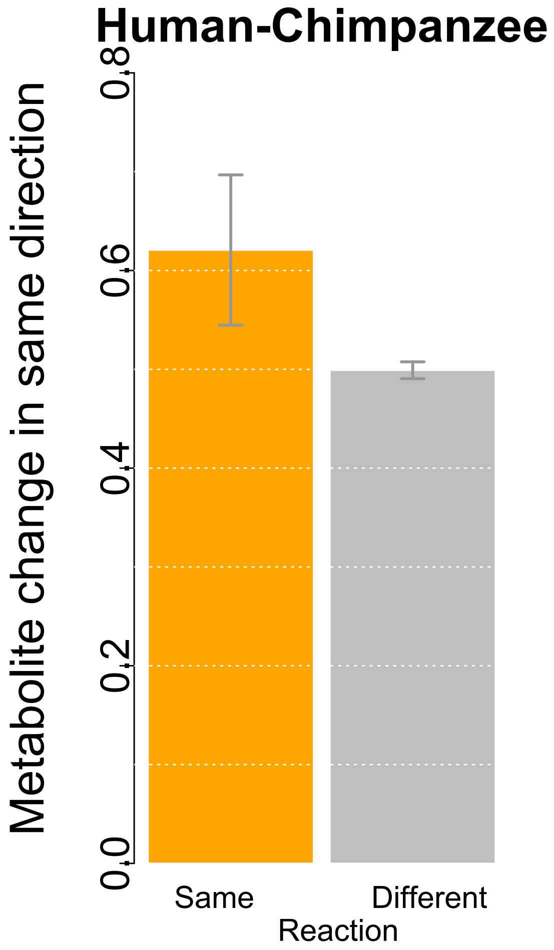

Figure S7. Similar to Figure 2D in the main text, but considering metabolites involved in the same reactions where at least one is DC between the species (and not necessarily both metabolites, as in Figure 2D).

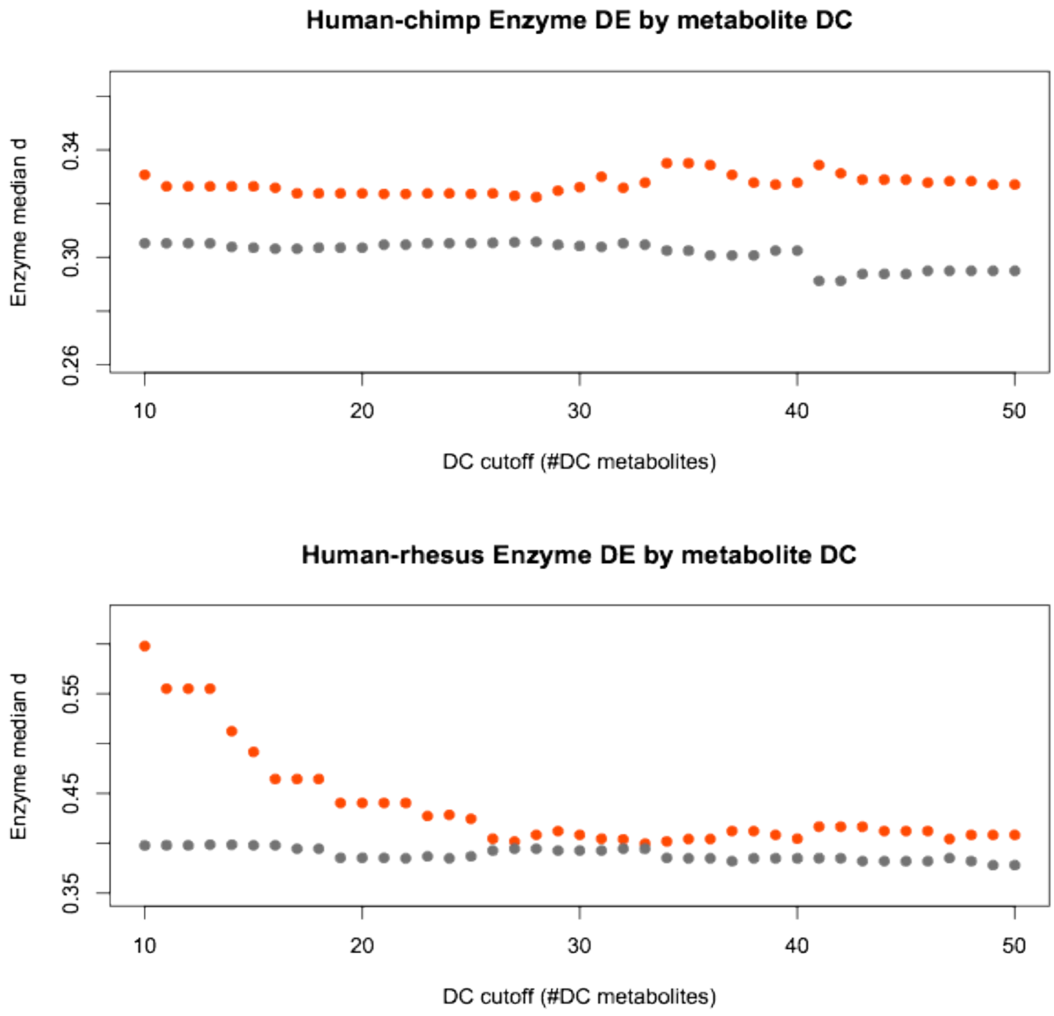

Figure S8. similar to Figure 3 (panels B and C) in the main text, with the *y*-axis showing the difference in estimated expression levels between human and chimpanzee for enzymes associated with DC (prange) and non-DC (grey) metabolites.

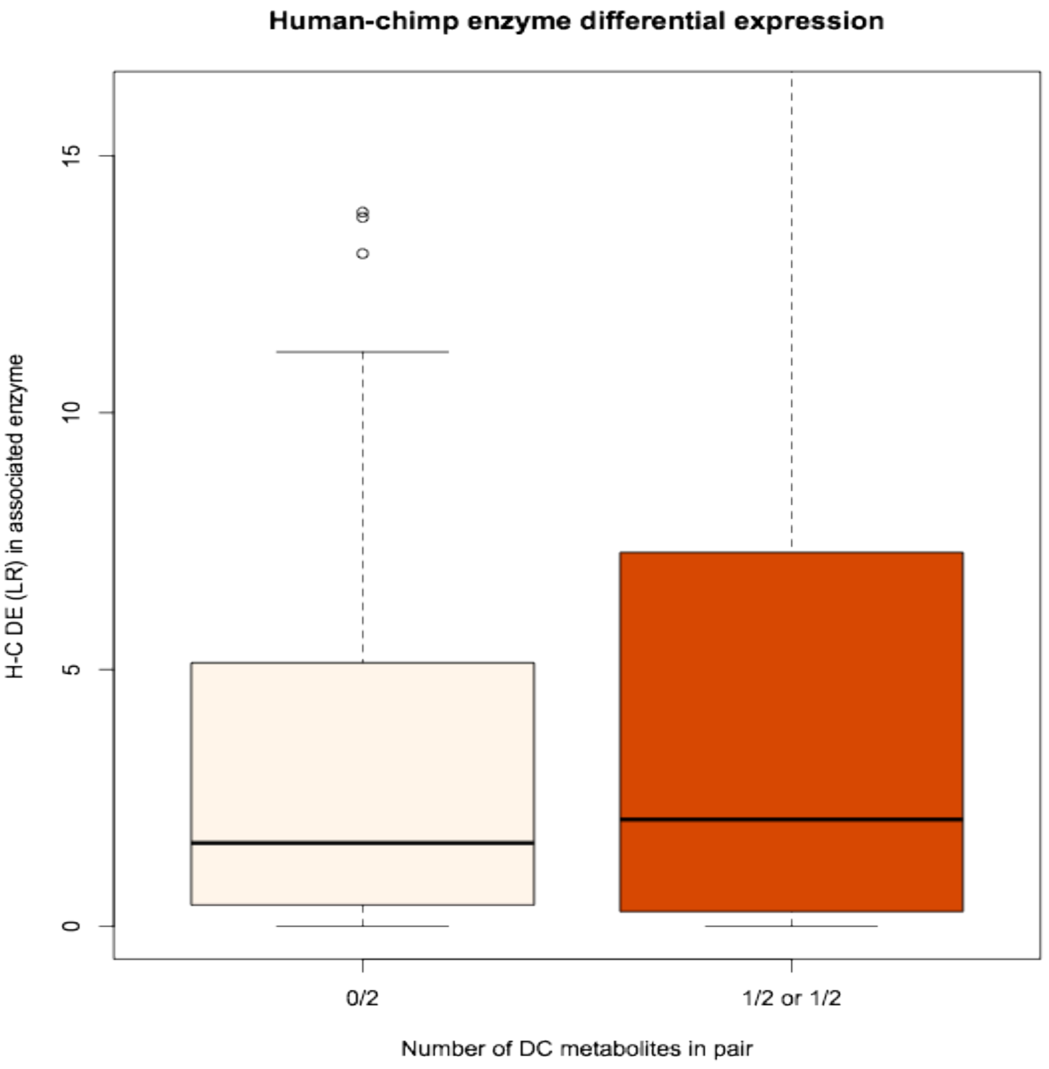

Figure S9. The number of differentially concentrated metabolites in a reaction is correlated with the level of differential expression of the associated enzyme. Enzyme LR (*y*-axis) for DE between human and chimpanzee, comparing enzymes controlling reactions in which none of the metabolites are DC (white), and reactions in which one or both metabolites are DC (orange).

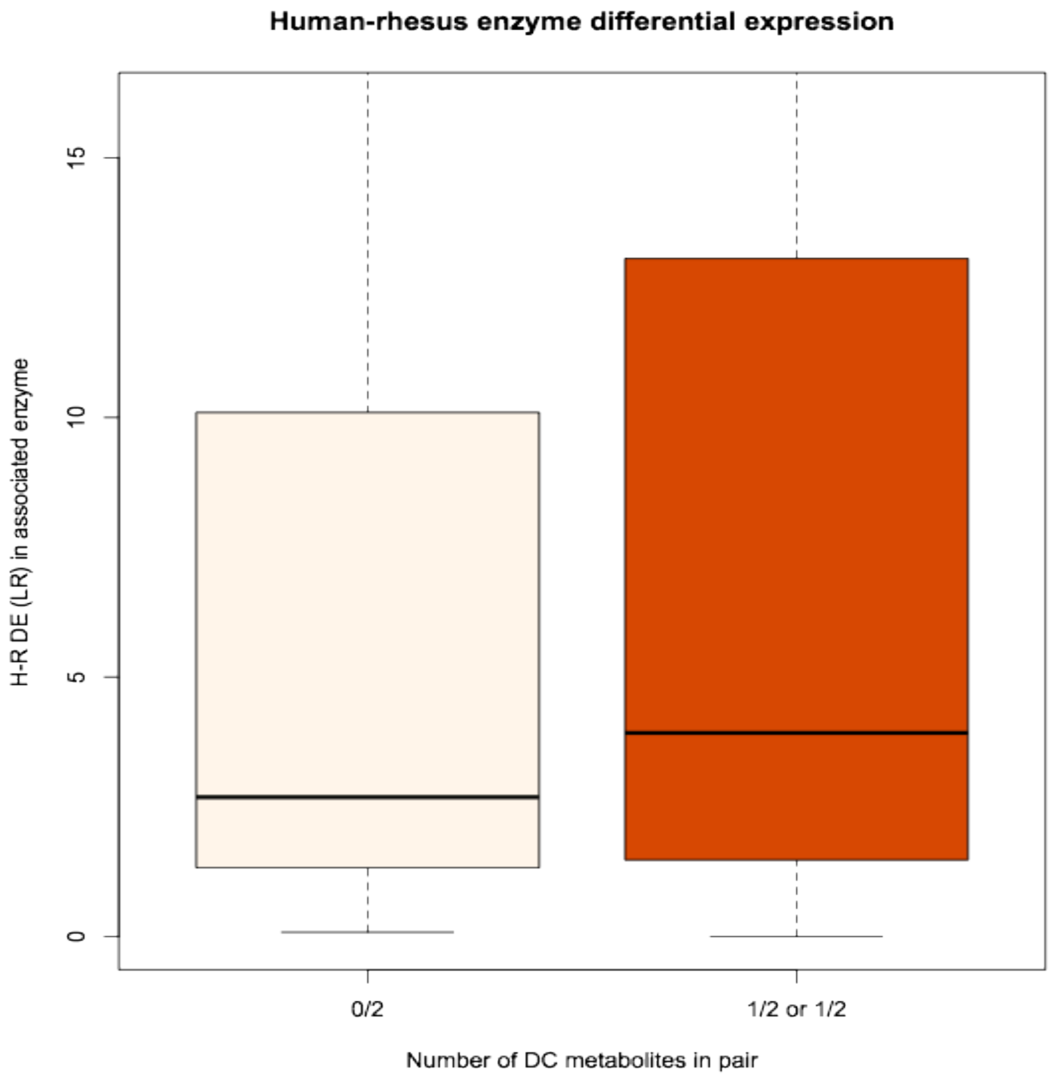

Figure S10. Similar to Figure S9, showing on the *y*-axis the LR for enzyme differential expression between human and rhesus macaque.
